# Age and gender patterns in emergency alarms and missions: a cross-sectional observational study

**DOI:** 10.1186/s12873-026-01479-x

**Published:** 2026-01-29

**Authors:** Kean Tang, Veronica Lindström, Markus Ådahl, Patrik Rydén

**Affiliations:** 1 Department of Mathematics and Mathematical Statistics, Umeå University, Linneaus väg 49, Umeå, 901 87 Sweden; 2 Department of Nursing, Umeå University, Linneaus väg 9, Umeå, 907 36 Sweden; 3 Umeå Centre for Gender Studies, Umeå University, Biblioteksgränd 6, Umeå, 907 36 Sweden

**Keywords:** Emergency prehospital care, Emergency medical services, Ambulance service, Priority-1 alarms, Response time, Statistical modelling

## Abstract

**Background:**

Emergency medical services play a key role in the healthcare system. An ageing population, economic challenges, and technological advances require emergency prehospital care to be flexible, efficient, and equal. We investigated emergency ambulance services in Västerbotten County, a sparsely populated region in northern Sweden, with the aim of understanding the spatio-temporal distribution of alarms and identifying factors that explain the response times of priority-1 alarms.

**Methods:**

We analysed 11,764 priority-1 alarms between 2022 and 2023 in Västerbotten County. The spatial and temporal distributions of the alarms were examined at various levels of aggregation, with a particular focus on age and gender. Response times were investigated together with its three components: dispatch time, preparation time, and travel time. Multivariate regression was used to analyse the components, considering six binary factors: age, gender, alarm location, season, time of the day, and day of the week.

**Results:**

The alarm incidence increased with age, with a sharp increase around 60–70 years of age, where the increase in alarm incidence was 13% (p $<$ 0.001) higher among men than women. The alarm incidence varied significantly over time, with the highest frequencies observed during daytime, weekdays, and winter season. The county’s median response time for priority-1 (MRT1) alarms was 14.6 minutes, with high variation between municipalities and even larger differences across rural, suburban, and urban districts. Elderly patients (60+) had 10% (p $<$ 0.001) longer MRT1 than younger patients (0–59). This resulted from elderly patients having longer dispatch and travel times, which could be attributed to the location of alarms. Interestingly, regardless of age, women had approximately 8% (p $<$ 0.001) longer dispatch times than men.

**Conclusion:**

From the age of 60, alarm incidence increases substantially, with a greater rise among men than women. A central finding of this study is that the EMS process times, including response times, are associated with the age and gender of the patients. These results are partly attributed to the spatial distribution of the alarms, but for the dispatch time the gender and age differences are arguably causal. Hence, an ageing population will result in more alarms and potentially longer missions, which will demand flexible and efficient EMS systems.

**Supplementary information:**

The online version contains supplementary material available at 10.1186/s12873-026-01479-x.

## Introduction

Emergency Medical Services (EMS) play a vital role in saving lives by offering prehospital care and transport to those in urgent need. The efficiency and effectiveness of these services are crucial to improving patient outcomes and ensuring equitable access to emergency services [1]. Despite their importance, EMS systems face challenges such as differences in the availability of emergency services and delayed response times, which are recognised as critical measures of service quality [2, 3]. Furthermore, the share of the population aged 60 years and older already exceeds 20% in the EU (21.6% as of January 2024 [4]), and Sweden’s population projections also indicate a marked ageing trend in the coming decades [5]. Global projections suggest that this segment will reach 22% by 2050 [6], implying greater demands on emergency services [7–9]. In addition, as informal family support networks shrink, more seniors may live alone and rely primarily on formal care, including ambulance transport [10–12]. In Sweden, the number of ambulance alarms has been rising over the past years, which has put pressure on EMS systems to deliver timely and effective care and has led to concerns about disparities in access to emergency services [13].

Previous research has identified several factors that significantly influence ambulance response times. Demographic characteristics such as patient age and gender show direct effects on response times. In Sweden, patients aged over 70 years were less likely to receive the highest dispatch priority than those aged 18 to 69 [12], while women with suspected stroke in the United States experienced longer median response times than men [14]. An Australian cohort study confirmed that age and gender predict delays, although temporal and geographic factors were stronger determinants [15]. Response times are consistently longer in rural areas compared to urban areas [16–18]. Slower responses were documented in northern Europe during rush hour congestion and weekend nights [19, 20], while adverse weather impedes ambulance travel in northern Norway and London [21, 22]. The increase in workloads without resource expansion also prolongs responses [19, 23]. Finally, socioeconomic disparities further aggravate the delays in both Sweden and France [24, 25].

Although previous studies have examined the impact of various factors on response times, little is known about how these factors influence the three components of response time, the dispatch time (i.e., the time from answered alarm call to resource dispatched), the preparation time (i.e., the time from resource dispatched to resource start mission), and the travel time (i.e., the time from resource start mission to arrival at scene), and the relative importance of these components. Analysing the components separately may be advantageous and reveal insights that enable optimisation of EMS systems, including placement and scheduling of resources.

The aim of the study is to gain an in-depth understanding of EMS systems, with a particular focus on the spatio-temporal distributions of the most urgent alarms, and how patterns of the corresponding process times depend on age and gender of the patient and time and location of the alarm.

## Methods

### Setting

Sweden is the largest country in northern Europe, with a population of 10.5 million in 2023. The Swedish healthcare system is mainly funded by taxes and is decentralised, with 21 regions responsible for providing healthcare services. The Swedish EMS system is part of the healthcare system and is responsible for providing emergency prehospital care to patients in need of emergency medical assistance. The ambulance service is coordinated by the emergency medical dispatch centre, usually operated by the publicly owned company SOS Alarm, which is commonly responsible for receiving emergency calls, dispatching ambulances, and providing medical advice to callers.

The study area of this work is Västerbotten County, located in the northern part of Sweden. Västerbotten has a Nordic climate with prolonged snowy winters and relatively mild summers. The county is sparsely populated with a population of 278,518 resistances, on an area of more than 54,664 square kilometres, and with a population density of 5.09 per km$^2$. Västerbotten faces geographical challenges that could affect alarm distributions, transportation logistics, and ultimately response times [26]. Although the county is sparsely populated, 72% of the population resides in urban areas and 28% in suburban and rural areas [27], where rural areas are characterised by low-density settlement and long distances between emergencies and ambulance bases [28]. This leads to a wide range of spatio-temporal variations in ambulance demand and potential disparities in access to care [29].

The county has a relatively old population, with 27% of the population older than 60 years, but there are large demographic differences between the county’s municipalities with an older population in the smaller inland municipalities and a relatively younger population in the cities close to the Baltic Sea.

EMS in Västerbotten County is provided by Region Västerbotten, which operates 15 road ambulance stations, one helicopter unit, and carries out around 30,000 missions per year, see Supplementary Table 1. The county’s EMS system is publicly financed and organised according to the Franco-German model [30].

### Data sources

Alarm and mission data of Västerbotten County for the period 2022–06-19 to 2023–06-18 were collected by Region Västerbotten. For each alarm, several variables were recorded including both alarm and mission data. The alarm data included information on the age and gender of the patients along with the time, location, and priority of the alarm. The mission data included information on the resource that carried out the mission and several process times, e.g., the response time, the dispatch time, the preparation time, and the travel time, see Supplementary Fig. 1.

To study the distribution of the alarms, the exact alarm coordinates were replaced by area data that included information on the district and municipality in which the alarms occurred. Sweden has been divided into around 6,000 districts, i.e., Demographic Statistical areas (DeSO), that can vary greatly in size, but where each district has between 700 - 2,700 inhabitants [27]. The districts are divided into three categories: type A, which includes sparsely populated rural areas, type B, which includes suburban areas, and type C, which includes urban areas. In Västerbotten County, 23%, 10%, and 67% of the population reside in districts of type A, B, and C, respectively [31].

Twelve of the municipalities in Västerbotten County have very small populations, ranging from 23,00–9,000 inhabitants, see Supplementary Table 1. To make the analyses robust, these small municipalities were merged into two geographical areas: the West area and the East area. The West area includes the municipalities Dorotea, Malå, Sorsele, Storuman, Vilhelmina, and Åsele, while the municipalities Bjurholm, Nordmaling, Norsjö, Robertsfors, Vindeln, and Vännäs create the East area, see Fig. 2. Henceforth, the analyses focus on the following five areas: the municipalities Lycksele (12,204 inhabitants), Skellefteå (76,219), Umeå (133,112), the East area (34,585) and the West area (22,398). All these regions have urban and rural districts, but the population density and ambulance staffing vary between the areas, see Supplementary Table 1.

### Statistical analysis

The annual number of alarms and the alarm incidence (i.e., the number of yearly priority-1 alarms per inhabitant) were calculated for the five areas and for the rural, suburban, and urban DeSO districts. Spatial analyses were performed on complete data and on alarm groups defined with respect to age, gender, alarm time, and alarm location. Pairwise alarm groups were compared using the two-sided binomial test when considering the number of alarms and the two-sided proportion z test when considering the alarm incidence, using the Python package SciPy version 1.11.2.

The temporal trends in the alarm data were investigated at three levels: seasonal, weekly, and hourly trends. The seasonal and weekly trend were fitted by LOWESS regression, using the function ‘lowess’, with the smoothing parameter ‘frac’ set to 0.1, in the Python package Statsmodels version 0.14.4. Differences in alarm frequencies between days of the week were analysed using the Chi-square test using the Python package SciPy version 1.11.2. The high-resolution hourly trend was explored in a bar plot, and the difference in alarm intensity between daytime hours (8:00–21:00) and nighttime hours (21:00–8:00) was analysed by the two-sided binomial test described above.

The four process times: response time, dispatch time, preparation time, and travel time, were initially analysed separately using descriptive analyses and pairwise analyses between alarm groups defined by age, gender, alarm time, and alarm location. The analyses focus on the median times, but occasionally the first quartile (Q1) and third quartile (Q3) quartiles were also considered. The Mann-Whitney U test was applied to test for median differences between the groups, using the Python package SciPy version 1.11.2. Additionally, Welch’s t-test was used to compare the mean differences in preparation time between the groups, using the Python package SciPy version 1.11.2.

Next, a multivariate model was applied to model the relationship between three process times: dispatch time, preparation time, and travel time, and a set of six binary factors: age (young vs old), gender (men vs women), alarm location (urban district vs suburban district or rural district), season (winter vs summer), day of week (weekday vs weekends), and time of day (daytime vs nighttime), see Supplementary Table 2. The levels of the factors were chosen after reasoning and visual inspection of the data. For age, the choice was to consider patients aged 0–59 years and patients older than 60 years, these groups have a similar amount of alarms and define a younger population group that is relatively healthy and an older group with more health problems. The seasonal factor distinguishes between a winter period (November to April), when snow and dark days are common, and a contrasting summer period (May to October) with long days and usually no snow. For the daily variation, we considered weekdays and weekends. The time of day was divided into a daytime group (8:00–21:00) and a nighttime group (21:00–8:00), based on visual inspection of the data, selecting the daytime hour period as the hours with the highest number of alarms. The model was fitted using bootstrapped multivariate linear regression [32]. The details of the algorithm and a GitHub link to the code are given in Appendix A. The modelling approach with binary factors and the dispatch, preparation, and travel times as the response variables enables an easy interpretation of the results, where the factors’ estimated coefficients describe the relative importance of the factors.

## Results

**Fig. 1:**
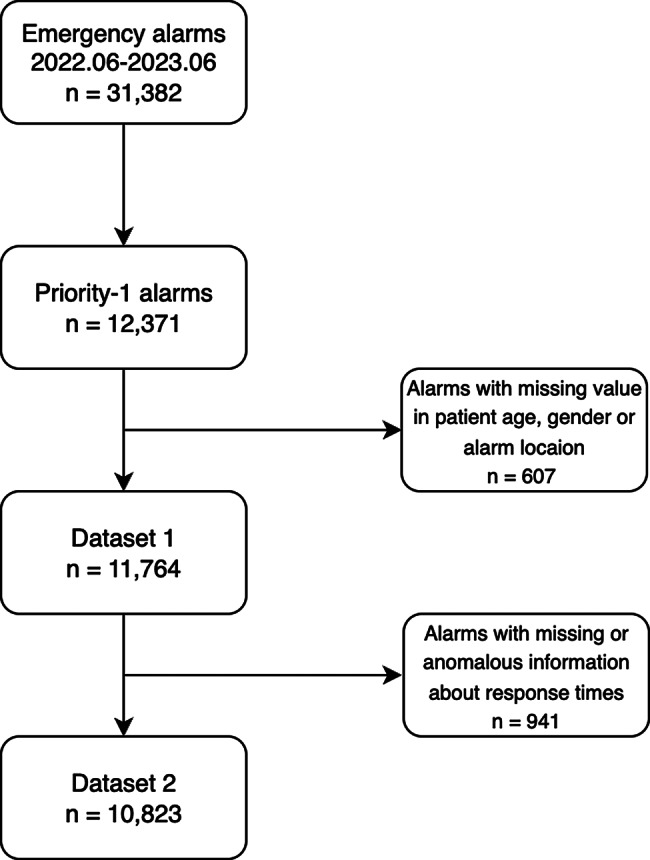
Selection of alarms

The selection of alarm data is shown in Fig. 1. This study focuses on priority-1 alarms and only includes data from missions carried out by road ambulances (12,371 alarms). In addition, missions with missing values in either age, gender or alarm location were excluded from further analyses. The data remaining after this initial filtering is referred to as dataset 1 (11,764 alarms). The results presented in Section “Spatial and temporal distribution of the alarms” are based on dataset 1. Dataset 1 included some alarms with anomalous travel times, that are likely to be errors, which were identified using road network data, see Appendix B. Alarms with anomalous travel times and alarms with no information about the response time, the dispatch time, the preparation time, or the travel time were excluded to construct dataset 2 (10,832 alarms). The results presented in Section “Spatial and temporal distribution of the alarms” are based on dataset 2.

### Spatial and temporal distribution of the alarms

**Fig. 2:**
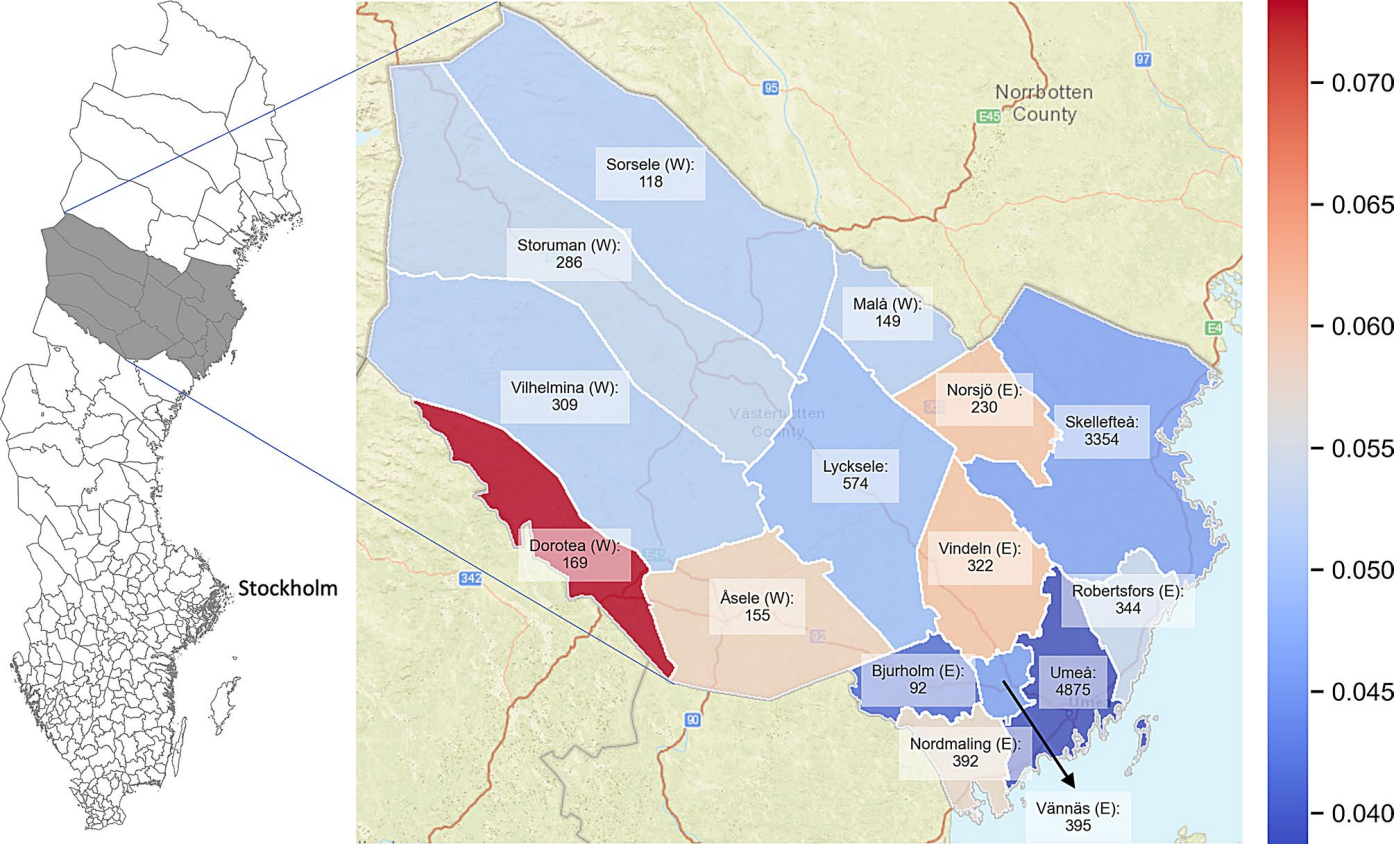
The number of priority-1 alarms in each municipality of Västerbotten County. The colours represent the corresponding alarm incidence. Västerbotten County comprises 15 municipalities, where the municipalities with low populations were merged into two areas: the East area, denoted by ‘(E)’ and the West area, denoted by ‘(W)’. Basemap: tiles © Esri—sources: Esri, DeLorme, NAVTEQ, USGS, Intermap, iPC, NRCAN, Esri Japan, METI, Esri China (Hong Kong), Esri (Thailand), TomTom, 2012

**Fig. 3:**
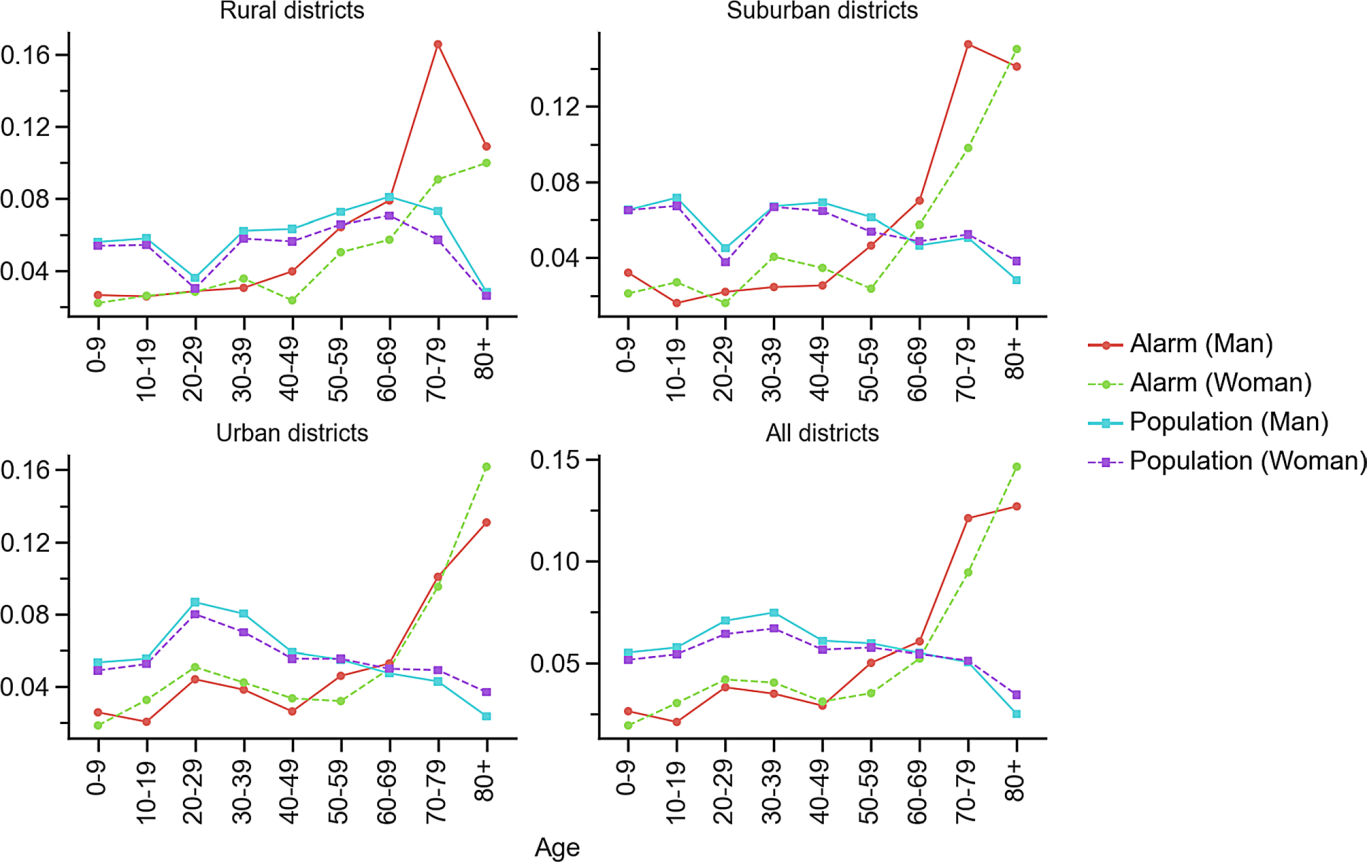
Fraction of priority-1 alarms by gender (men green solid line; women red dashed line) across age groups in all districts and aggregated DeSO districts: rural districts (A), suburban districts (B), and urban districts (C). The corresponding population distribution is shown by age group (men blue solid line; women purple dashed line). In both cases, the male and female proportions sum to 1

Most priority-1 alarms occurred in Umeå (4,875), followed by Skellefteå (3,354), see Fig. 2. Sixty-seven percent of the alarms occurred in urban districts (i.e., type C districts), 23% in suburban districts (type B), and 10% in rural districts (type A), see Supplementary Table 3. Women accounted for 49% of all alarms, and the median patient age was 59.8 years for both men and women. Elderly patients (60+) represented 60% of the alarms in the region, although only 27% of the population were elderly; see Table 1. The number of alarms per age group was similar for patients younger than 50 years, but increased sharply for older patients. Interestingly, this increase was more pronounced among men, see Fig. 3 and Supplementary Fig. 2.

**Table 1:** Population, alarm, and incidence data

Area	Population			Priority-1 alarms			Alarm incidence				
	All	% Women	% 60+	All	% Women	% 60+	All	Men	Women	0–59	60+
East area	34,585	0.49	0.33	1,787	0.49	0.63	0.051	0.051	0.051	0.028	0.098
Lycksele	12,204	0.49	0.31	574	0.44	0.67	0.047	0.051	0.042	0.023	0.099
Skellefteå	76,219	0.48	0.30	3,354	0.48	0.61	0.044	0.044	0.044	0.024	0.092
Umeå	133,112	0.50	0.22	4,875	0.52	0.57	0.037	0.035	0.038	0.020	0.093
West area	22,398	0.48	0.38	1,186	0.43	0.63	0.053	0.058	0.048	0.032	0.088
All areas	278,518	0.49	0.27	11,764	0.49	0.60	0.042	0.042	0.042	0.023	0.093

Population, number of priority-1 alarms, and alarm incidence for different areas and demographic groups, where % 60+ denotes the proportion of citizens aged 60 years and older

**Fig. 4:**
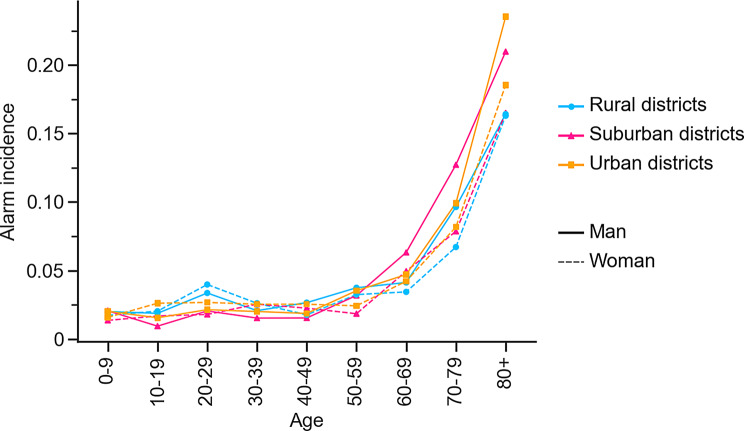
The alarm incidence for men (solid line) and women (dashed line) in aggregated DeSO districts: rural districts (A; blue), suburban districts (B; pink), and urban districts (C; yellow)

Similar trends were observed when the alarm incidence was investigated. The overall alarm incidence was 0.043 for the total population, 0.024 among individuals aged 0–59 years, and significantly higher among those aged 60 years and older (0.093, p $<$ 0.001). Alarm incidence began to rise around the age of 60, see Fig. 4. For the aggregated population, this increase was significant (p $<$ 0.001), ranging from an incidence of 0.018 for individuals aged 0–9 to 0.274 among those older than 90 years, see Supplementary Table 4.

Notably, the gender difference among the elderly increased with age from a difference of 0.006 (p $<$ 0.01) for the age group 60–69 to 0.060 (p $<$ 0.001) for citizens older than 90 years, see Supplementary Table 5.

**Fig. 5:**
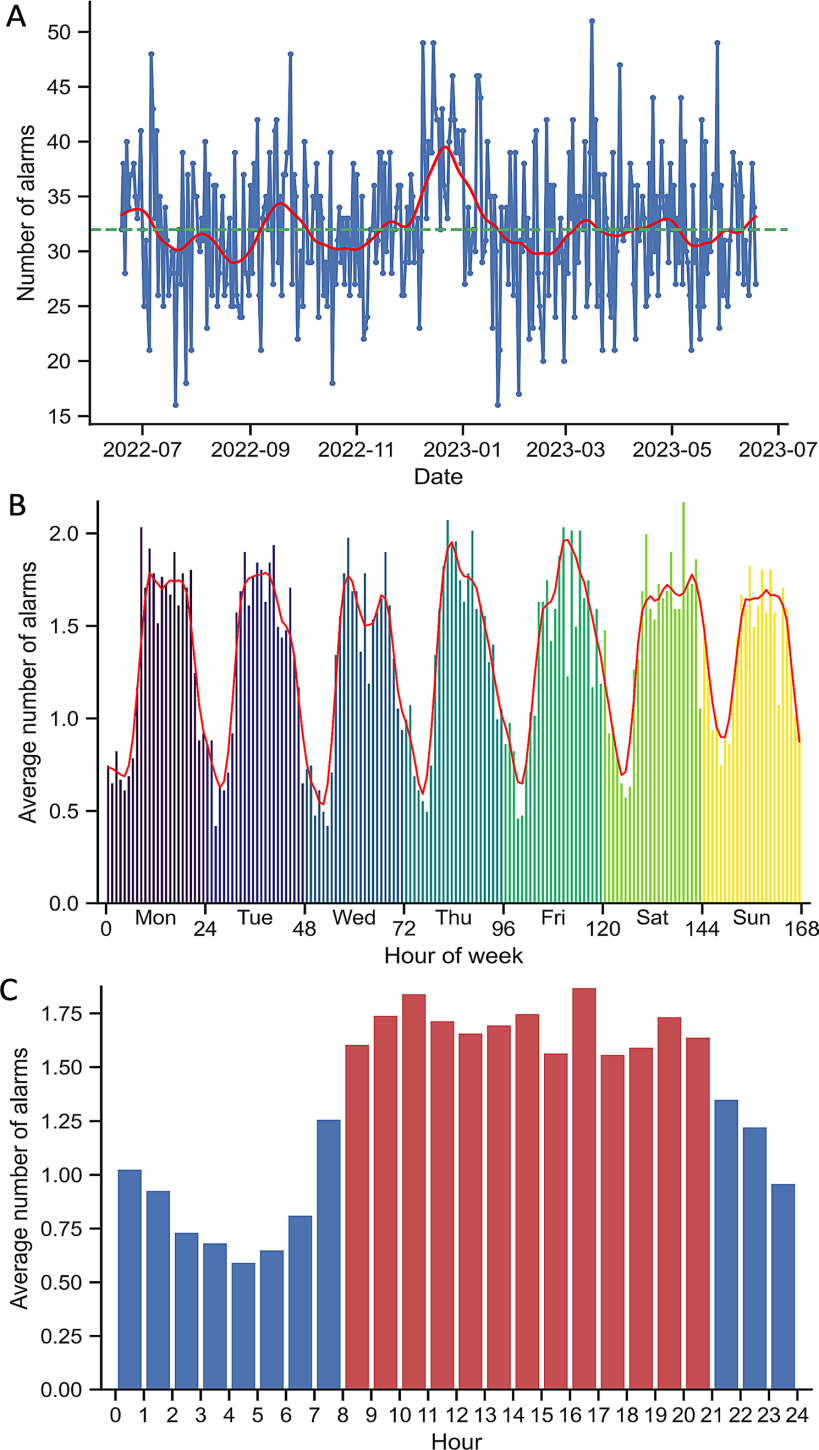
Figure **A** shows the daily number of priority-1 alarms in Västerbotten County during the study period, together with an estimated seasonal trend (red line) and the median number of alarms (green dashed line). Figure **B** shows the average number of alarms per hour during a week, together with an estimated weekly trend (red line). Here the average number of alarms per day of the week were 32.2 (Monday), 32.0 (Tuesday), 30.2 (Wednesday), 32.7 (Thursday), 32.5 (Friday), 34.2 (Saturday), and 32.4 (Sunday). Figure **C** shows the average number of alarms per hour during a day, where the daytime hours are coloured red and the nighttime hours are coloured blue

Several temporal trends were observed in the alarm data, including a seasonal trend, a weekly trend, and a highly pronounced hourly trend. There was a seasonal peak with most alarms in December and January, but the daily variation was high, see Fig. 5A. The variation between the days of the week was significant (p $<$ 0.05), ranging from an average of 30.2 alarms on Wednesdays to 34.2 alarms on Saturdays, see Fig. 5B. The most pronounced temporal pattern was observed in the hourly distribution of alarms, with 68.2% of alarms that occur during daytime hours (8:00–21:00). During this period, the average number of alarms per hour was 1.69, compared to 0.93 alarms per hour at night (p $<$ 0001), see Fig. 5C.

### Analysis of response time and associated process times

The median response time for priority-1 alarms (MRT1) in Västerbotten County was 14.6 minutes, with the first and third quartiles being 9.9 and 23.0 minutes, respectively. The relatively long response times can be attributed to Västerbotten County’s sparse population, limited number of ambulance stations, and long travel distances. However, there were significant differences between the five areas, with MRT1 ranging from 11.5 minutes in Lycksele to 22.4 minutes in the West area, see Supplementary Table 6.

The response time is the sum of three process times: the dispatch time, the preparation time, and the travel time(travel time to scene). The distributions of the dispatch, preparation and travel times were highly skewed with long right tails, see Supplementary Fig. 4. The skewed distribution of the dispatch time was partially explained by occasional queuing, where the dispatch of missions was delayed due to all ambulances being occupied. Unfortunately, the data does not include explicit information on queueing times. The skewed distribution of the preparation times was partially attributable to the use of on-call readiness staff at some stations during nighttime, primarily in municipalities with relatively small populations. Finally, the right tail in the distribution of travel times was mainly explained by alarms in rural areas far from ambulance stations.

The differences in MRT1 between areas were mainly explained by differences in travel times, e.g., the median travel times varied between 5.1 minutes in Lycksele and 11.5 minutes in the West area. A notable observation is that these differences almost disappear when rural, suburban, and urban districts are analysed separately, suggesting that demographic factors account for the travel time differences between areas, see Supplementary Table 6. Thus, in Västerbotten County, MRT1 values do not depend on the area in which a person lives, but rather on the type of district in which they live. Henceforth, in Västerbotten County, response times are mainly determined by the type of district in which one lives than by the specific area. This can be explained by the fact that all the county’s ambulance stations were in urban districts, usually within the municipalities’ largest cities, and that all but one municipality had a station.

#### Differences with respect to age and gender

Elderly patients, here defined as patients older than 60 years of age, had longer response times than younger patients aged 0–59 years. The MRT1 for the elderly and younger patients were 15.2 and 13.8 minutes, respectively, with a difference of 1.4 minutes (p $<$ 0.001), see Supplementary Table 7. This difference was primarily due to longer dispatch and travel times among elderly patients. No significant age differences were observed for preparation times. The median dispatch times for elderly and younger patients were 3.9 and 3.1 minutes, respectively, with a difference of 0.8 minutes (p $<$ 0.001). The median travel time for elderly patients was 8.9 minutes, which was 0.8 minutes longer than for younger patients (p $<$ 0.001), see Supplementary Table 7. An explanation for the differences may be demographic differences. Therefore, the rural, suburban, and urban districts were analysed separately.

For travel times, no significant differences were observed in suburban and urban districts, while a 1.5 minute difference (p $<$ 0.01) was observed in rural districts, see Supplementary Table 7. This implies that the travel time difference was partially determined by demographic differences, with the younger population having a larger fraction of alarms within urban districts than the elderly population, see Supplementary Table 3. A further investigation revealed that the age related difference in travel time observed in rural areas was explained by the locations of the alarms. The estimated travel time, i.e., the predicted ambulance travel time assuming the fastest route between the starting position and the pickup location while adhering to speed limit, was on average 2.2 minutes longer for the elderly population. Thus, the entire age 1.5-minute difference in travel time within rural districts could be attributed to the spatial distribution of the alarms, see Supplementary Table 7. Therefore, elderly patients had longer travel times than younger patients, mainly due to the location of the alarms.

Age related differences in dispatch time were highly significant (p $<$ 0.001) in all types of districts, see Supplementary Table 7. Interestingly, dispatch times increased linearly with age for both men and women, see Supplementary Fig. 5. For men, the median dispatch time rose from 2.4 minutes in the 0–9 age group to 4.0 minutes for those over 90 years of age. The corresponding values for women were 2.7 and 4.2 minutes, respectively.

The MRT1 for men was 0.7 minutes longer than for women (p $<$ 0.001), but this difference was only significant for the elderly patients, see Supplementary Table 8. A closer examination of the travel times reveals that men had a 1.0 minute longer travel time than women (p $<$ 0.001), see Supplementary Table 8. This gender difference was only significant for elderly patients in rural districts, but a similar difference was also observed for the estimated travel times, suggesting that the difference could be explained by the distribution of the alarms, see Supplementary Table 8. Since most alarms occur close to the residences of patients, this suggests that elderly men in rural districts tend to live in more remote locations than elderly women.

Women had a 0.3 minute longer dispatch time than men (p $<$ 0.001), see Supplementary Table 8. This difference was 0.43 minutes (p $<$ 0.001) for young patients and 0.30 minutes (p $<$ 0.001) for elderly patients. Furthermore, gendered dispatch time differences were observed for all types of districts and age groups, suggesting that these differences are not likely to be explained by demographic gender differences, see Supplementary Table 8.

#### Multivariate regression analysis

The multivariate analysis complements the univariate analysis in the previous section. The analysis was based on dataset 2 including 10,823 priority-1 alarms, see Section “Data sources”.

Multivariate linear regression was used to simultaneously model dispatch time, preparation time, and travel time as functions of six binary variables: age, gender, alarm location, season, time of day, and day of the week, see Supplementary Table 2. The use of binary variables allowed us to compare how the factors influence process times by examining their estimated coefficients. Moreover, the analyses in the previous section focused on median values, while the coefficients in the regression analyses relate to average effects. The mean response time for priority-1 alarms in Västerbotten County was 22.0 minutes, which is considerably higher than the MRT1 of 14.6 minutes, which was explained by the skewed distributions of the response times, see Supplementary Fig. 4.

Overall, the main results of the univariate and multivariate analyses resemble each other, but the effect size and p-values differ due to different key measures (mean and median), slightly different data, and different complexity of the analyses.

As expected, travel times were determined primarily by the place of residence. On average, alarms occurring in rural or suburban districts had response times that were 13.3 minutes longer (p $<$ 0.001) than in urban districts. Three additional factors affected travel times: men had, on average, 0.76 minute longer travel times than women (p $<$ 0.001), elderly patients had 0.62 minute longer travel times than those aged 0–59 (p $<$ 0.001), and travel times during November-April were, on average, 0.70 minutes longer than during May-October, see Table 2. The age and gender results correspond with the univariate analysis, and the seasonal difference may be explained by short chilly days, and often snowy conditions.

**Table 2:** Summary of multivariate regression analysis

Factor	Response	Estimated coefficient	95%-confidence interval	
			Lower bound	Upper bound
Intercept	Dispatch time	4.09***	3.88	4.31
	Preparation time	1.85***	1.79	1.92
	Travel time	21.11***	20.61	21.62
Age	Dispatch time	0.61***	0.46	0.76
	Preparation time	0.09***	0.05	0.14
	Travel time	0.62***	0.27	0.96
Gender	Dispatch time	0.28***	0.13	0.42
	Preparation time	−0.06**	−0.10	0.01
	Travel time	−0.76***	−1.09	0.43
Alarm location	Dispatch time	−0.32***	−0.48	−0.17
	Preparation time	−0.23***	−0.27	−0.18
	Travel time	−13.28***	−13.64	−12.92
Season	Dispatch time	0.22**	0.07	0.36
	Preparation time	0.11***	0.06	0.15
	Travel time	0.70***	0.37	1.03
Day of the week	Dispatch time	−0.25**	−0.41	−0.10
	Preparation time	0.08***	0.03	0.13
	Travel time	−0.06	−0.42	0.31
Time of the day	Dispatch time	0.07	−0.09	0.22
	Preparation time	−0.77***	−0.82	0.72
	Travel time	0.04	−0.33	0.40

Predicted coefficients together with corresponding p-value and 95%-confidence interval forbootstrapped multivariate regression with the response variables dispatch time, preparationtime, and travel time and the six binary variables age, gender, alarm location, season, day ofthe week, and time of the day. The number of stars corresponds to different p-values: * (p <0.05), ** (p < 0.01), and *** (p < 0.001)

The preparation times were affected by the time of day and the location of the alarm. The preparation times were, on average, 0.77 minutes longer (p $<$ 0.001) during the evening and night hours (21:00–8:00) compared to daytime hours (8:00–21:00), and the rural and suburban districts had, on average, 0.23 minutes longer (p $<$ 0.001) preparation times than the urban districts. The observed age and gender related differences in preparation time could be explained by variations in the use of on-call readiness during night hours across different stations. To explore this further, we compared preparation times between municipalities with stations operating under on-call readiness at night, hereafter referred to as small municipalities (i.e., municipalities in the East and West area except Nordmaling, Vännäs and Robertsfors), and the other municipalities in the county with 24/7 on-site readiness referred to as large municipalities. When the smaller and larger municipalities were analysed separately, no significant age or gender related differences in preparation time were observed, see Supplementary Tables 9 and 10. These findings support the assumption that the age and gender related differences in preparation time are primarily explained by the use of on-call readiness.

The dispatch time was influenced by all factors considered except the time of day. The most crucial factor here was age for which the dispatch times were 0.61 minutes longer (p $<$ 0.001) for the elderly patients compared to the younger group of patients. Women had, on average, 0.28 minutes longer (p $<$ 0.001) dispatch times than men. Furthermore, there was a seasonal effect, with longer dispatch times during the winter months (0.22 minutes, p $<$ 0.01), and a weekend effect, with shorter dispatch times on Saturdays and Sundays (0.25 minutes, p $<$ 0.01). Additionally, there was a residential effect, where alarms occurring in rural and suburban districts had, on average, 0.32 minute longer dispatch times than in urban districts (p $<$ 0.001). The effects of age and gender are in agreement with the univariate analysis, while the residential effect was not easily observed in the univariate analysis.

## Discussion

The results suggest that the alarm incidence increases with age, with a sharp rise around 60 to 70 years, which is consistent with previous studies [33–35]. Interestingly, the increase in alarm incidence was higher among men than among women. Furthermore, analyses suggest that response times for older patients are longer than for younger patients. Hence, an ageing population will result in more alarms and potentially longer missions. The value of the results is that they enable predictions on how EMS will be impacted by future demographic changes. According to data from Statistics Sweden [5], the proportion of elderly residents in Västerbotten County is expected to increase in the coming decades, while the overall population will remain at a level like that of 2023. If the alarm incidence rates presented in Table 1 remain constant, this demographic shift is projected to lead to a 6.7% increase in priority-1 alarms by 2030 and an 18.4% increase by 2050, compared to 2023. An approach to assessing how these demographic changes may impact response times and strain on the EMS system is through large-scale data-driven simulations [36]. Predicting the effects of anticipated demographic changes will provide valuable insight into the strategic planning and organisation of EMS systems.

A central finding of this study is that the EMS process times, including response time, are associated with the age and gender of the patients. These results are partly attributed to the spatial distribution of the alarms, but for the dispatch time the gender and age differences are arguably causal.

Dispatch times increase linearly with age. A comparison between the youngest patient group (children aged 0–9) and patients over 90 years of age shows that the latter group experiences 60% longer median dispatch times. The data do not provide a clear explanation for this finding, but it is reasonable to assume that it may be related to three factors: communication challenges, variations in medical conditions, and differences in prioritisation. Here, dispatch time is defined as the time it takes from answering the alarm call to dispatching the alarm to an available resource. In the region, the call taker converses with the caller, using standardised protocols, and assigns the initial priority to the alarm. The dispatcher monitors the call and can dispatch the alarm to an appropriate unit at any time. Typically, both the call taker and the dispatcher have limited medical training. If the ‘most suitable ambulance’ for the mission is not available at the time when the dispatcher is ready to dispatch the ambulance, the dispatcher has to wait until the resource is available. Hence, the observed dispatch time is the sum of the decision time, i.e., the time it takes for the dispatcher to decide which is the most suitable ambulance for the mission, and a possible queue time. The time for decision-making is influenced by the caller’s ability to communicate, whether the caller is the patient or another person on scene, and by the underlying cause of the alarm. Children typically do not make their own emergency calls, and among adult patients, elderly individuals often suffer from complex multiple health conditions that can make communication more difficult. Furthermore, we speculate that dispatchers and call takers may perceive calls involving younger patients, particularly children, as more urgent, especially when the symptoms are vague. A Danish study [37] reported that emergency calls for patients under 15 years of age were often characterised by non-specific symptoms, and that the most common response was an immediate ambulance dispatch with lights and sirens.

Women had approximately 8% longer dispatch times than men, regardless of age. Again, this could be due to communication difficulties and gender differences in the underlying causes of the alarms or symptom presentations during the call. [38] provide concrete evidence of gendered differences in EMS dispatch prioritisation. Their study of 27,805 calls to Swedish EMDCs found that males were associated with a higher likelihood of receiving a high-priority ambulance dispatch, even though women often report conditions (e.g., vague or unclear symptoms, psychiatric problems) that can complicate assessment of the call and triaging. This indicates a persistent gender bias in prioritisation, where male patients may be perceived as requiring more urgent responses. From an intersectional point of view, gender bias in prioritisation may also interact with other social determinants such as age, socioeconomic status, and ethnicity. For example, an elderly woman with limited social support in a rural setting can experience compounded delays due to both gendered perceptions of urgency and weak social support.

In considered areas, a large proportion, often a majority, of the population, lives in urban districts. This presents a challenge for planning and organisation: How should the EMS system be structured to effectively meet the demands? It could be argued that all citizens should have access to emergency aid within a reasonable time, but this cannot be secured individually. A more practical approach is to ensure that residents of a defined area, such as a county, municipality, or city, have, as a group, access to EMS of reasonable quality. In Västerbotten County, the major differences in MRT1 are not between defined areas, but between rural, suburban, and urban districts within those areas. This can be explained by three factors: most areas have the majority of their population living in urban districts; most urban districts are located close to an ambulance station; and the median measure MRT1 is largely determined by alarms occurring in urban districts, since most residents live there. For example, comparing the MRT1 values between sparsely populated Lycksele (2.21 inhabitants/km$^2$) and Umeå (56.47 inhabitants/km$^2$) reveals that Umeå has a median MRT1 that is 2 minutes longer than in Lycksele. However, in Lycksele, the MRT1 for rural and urban districts are 9.87 minutes and 30.30 minutes, respectively, while the corresponding values for Umeå are 12.13 and 22.82 minutes. This example illustrates the limitations of presenting only area-level MRT1 values. To improve EMS performance reporting, these limitations can be addressed either by also considering MRT1 values at a more refined scale, in our case districts, or by additional descriptive measures such as mean, trimmed mean, first quartile, and third quartile of the response times. The choice of key measures will have a direct impact on strategic decisions regarding the allocation resources, including scheduling resources and location of stations.

Predicting the future length of missions in the context of an ageing population is a complex task. It requires not only forecasting changes in dispatch time, preparation time, and travel time to scene but also accounting for other dynamic processes, such as the development of queues. We argue that an ageing population is likely to result in longer dispatch times. Moreover, an increase in the number of alarms could lead to more frequent and prolonged queues unless sufficient resources are added to the prehospital system. However, it remains unclear whether an ageing population will also lead to longer travel times. At present, elderly individuals are overrepresented in rural districts, which naturally contributes to longer travel times. Assuming that the spatial distribution of alarms across rural, suburban, and urban areas remains constant for both younger and older patients, an ageing population would indeed result in longer travel times, but this assumption is uncertain and may not hold in the future. Shifts in mobility patterns, such as migration from rural to urban areas for work or education, or from city centres to suburban or rural areas for improved quality of life, are likely to directly influence travel times.

An ageing population that results in more and potentially longer missions will require more resources and a more effective use of resources to maintain current EMS quality. Deploying mobile ambulance units that can be relocated based on demand patterns and working with temporal staffing adjustments may be a cost-effective approach, although it comes with challenges. With the majority of future patients expected to be elderly, it is crucial to implement targeted health interventions and community support programmes aimed at reducing the incidence of emergency situations among this group [39]. Addressing gender disparities will also require tailored strategies, which could include targeted training for personnel responsible for dispatching alarms. Technological enhancements and data-driven decision support can further improve efficiency [36, 40]. Implementing advanced GIS and real-time monitoring systems can optimise dispatch decisions, thereby reducing response times. Continuous analysis of spatial and temporal data can enable dynamic resource allocation to meet changing demands and improve overall service efficiency.

## Limitations and future research

The study has limitations that should be taken into account when interpreting the results. First, it focuses on priority-1 alarms, since their EMS response is crucial for survival and less urgent alarms are more difficult to analyse. Less urgent missions are often queued due to lack of available ambulances. Unfortunately, the data do not include specific information on how long ambulances were queued, which poses a challenge when modelling dispatch and preparation times.

Second, the considered priority variable represents the final priority set by the dispatcher before the ambulance arrives on scene. Hence, the data do not provide information on earlier or later prioritisation, e.g., initial prioritisation, triage by ambulance crews, and subsequent hospital assessments. How priority is determined may affect the results, including the estimated effects of age and gender on MRT1. In particular, EMDCs often assign higher urgency than ambulance crews, resulting in potential over-triage and more urgent alarms. Additionally, unmeasured variability may arise from the interpretation of the dispatch information by ambulance crews. Ambulance crews may integrate their contextual judgement and professional experience into their responses, which can influence driving behaviour, use of blue lights and sirens, and overall response times, regardless of the dispatched priority level.

Furthermore, the study does not include downstream EMS processes, e.g., time at scene, driving time to destination, and handover time at hospitals. Analyses of these processes may contribute to a better understanding of how prehospital emergency care will be affected by an ageing population. Preliminary analyses suggest that time on scene is age dependent. This may affect mission duration, ambulance availability, and indirectly response times.

Finally, the study covers only a single year. Longitudinal analyses over multiple years would allow examination of trends in alarm patterns, prioritisation practices, and EMS operational performance.

## Conclusions

We investigated the EMS system in Västerbotten County based on data from 2022 to 2023 using two principal different analysis approaches: univariate analyses based on the median measures and multivariate analyses based on average measures. In addition, the analysis of the response time was complemented with analyses of its components, i.e., the dispatch time, the preparation time, and the travel time. However, the study only considers the most urgent alarms and does not consider downstream EMS processes, e.g., time at scene, driving time to destination, and handover time at destination. Hence, the study design may not capture the broader EMS trends. Conducting longitudinal studies over an extended period can help identify long-term trends and the impact of implemented policies on ambulance service efficiency. Such studies would provide valuable data on the evolution of service patterns and the effectiveness of interventions over time. Moreover, the absence of detailed patient information, such as medical history, clinical outcomes, socioeconomic data, is a limitation. Future research could explore the integration of patient health records with ambulance dispatch data to provide a more comprehensive understanding of the patient population and their healthcare needs.

Our study contributes to the ongoing discussion on EMS demand management, providing insights that can guide both local and global efforts to improve emergency medical care delivery. Future research should focus on exploring innovative solutions to the identified challenges, with a continued emphasis on reducing response time disparities and optimising ambulance service strategies to meet the evolving needs of the population.

## Electronic supplementary material

Below is the link to the electronic supplementary material.

Supplementary Material 1

## Data Availability

The datasets analysed during the current study are not publicly available, but can be requested from the corresponding author upon reasonable request.

## Abbreviations

EMS: Emergency medical services
LOWESS: Locally weighted scatterplot smoothing
MRT1: Median response time of priority-1 alarms
EMDC: Emergency medical dispatch centres
GIS: Geographic information system
