# Supplementary material for: Age and gender patterns in emergency alarms and missions: a cross-sectional observational study
Source: BMC Emerg Med. 2026 Jan 29;26:60. doi: 10.1186/s12873-026-01479-x (PMC12924591; doi:10.1186/s12873-026-01479-x)
Supplement: Supplementary file 1 — Supplementary Material 1 [file 12873_2026_1479_MOESM1_ESM.pdf]

Supplementary Materials

Appendix A Algorithm for Multivariate linear regression with bootstrapping

The multivariate linear regression model can be expressed as

$$\mathbf{Y} = \mathbf{X}\boldsymbol{\beta} + \boldsymbol{\epsilon},$$

where $\mathbf{Y}$ is an $n \times r$ matrix of response variables, $\mathbf{X}$ an $n \times p$ design matrix of predictors (including an intercept), $\boldsymbol{\beta}$ a $p \times r$ matrix of coefficients, and $\boldsymbol{\epsilon}$ an $n \times r$ matrix of residuals with $\epsilon_i \in \mathbb{R}^r$ having mean zero and positive definite covariance matrix Σ. Here, n is the number of observations, p the number of predictors, and r the number of response variables. Each column of $\mathbf{Y}$ represents a separate regression on the same predictors in $\mathbf{X}$. The least squares estimator is

$$\hat{\boldsymbol{\beta}} = (\mathbf{X}^T\mathbf{X})^{-1}\mathbf{X}^T\mathbf{Y}.$$

An example of multivariate linear regression with bootstrapping is given in https://github.com/KeanTanG/Multivariate-Linear-Regression-with-Bootstrapping. The algorithm is as follows:

Algorithm 1 Bootstrapped multivariate linear regression

```
Fit the model to the original dataset {X, Y} to obtain estimated coefficients β̂.
Compute residuals ε = Y − Xβ̂.
for i = 1 to B do                                  ▷ B is the number of bootstrap samples
    Sample n residuals with replacement from ε to obtain bootstrap residuals ε*.
    Form bootstrap responses Y* = Xβ̂ + ε*.
    Fit the model to {X, Y*} to obtain bootstrap coefficients β*.
    Store the bootstrap coefficients β* for inference.
end for
Compute statistics (e.g., confidence intervals) for β from the distribution of β*.
```

Appendix B Filtering rules for travel times

To ensure data quality, implausible travel times were excluded according to the following rules:

1. Alarms with time at scene (TS) shorter than 10 seconds were removed, as such values likely reflect recording errors.
2. Alarms with travel time (TT) exceeding the estimated travel time (ETT) when TS was shorter than 60 seconds were removed.
3. Alarms with TT shorter than half of ETT were excluded.
4. Alarms with TT longer than four times ETT when ETT exceeded 180 seconds were removed.
5. Alarms with TT exceeding 5000 seconds and differing from ETT by more than 20% of TT were excluded.

where estimated travel times were computed with road network data and the function 'shortestpath' in MATLAB R2022a. The road network was obtained from the 2019 release of the Swedish National Road Database (NVDB) provided by Trafikverket. The network was represented as a graph, where intersections or road endpoints were defined as nodes and road segments as edges, with associated attributes such as length and driving speed.

Formally, alarms were discarded if any of the following conditions held:

1. $\mathrm{TS} < 10$,
2. $\mathrm{TT} > \mathrm{ETT} \ \wedge \ \mathrm{TS} < 60$,
3. $\mathrm{TT} < 0.5 \times \mathrm{ETT}$,
4. $\mathrm{TT} > 4 \times \mathrm{ETT} \ \wedge \ \mathrm{ETT} > 180$,
5. $\mathrm{TT} > 5000 \ \wedge \ |\mathrm{TT} - \mathrm{ETT}| > 0.2 \times \mathrm{TT}$.

Supplementary Table 1: Population, land area (km^2) and population densities (people/km^2) of the municipalities and areas in Västerbotten County, together with available prehospital resources including number of stations and road ambulances. The data were collected in November 2023.

Area/Municipality	Population	Land area	Population density	Stations	Ambulances
East area	34,585	8,728	3.96	5	5
Bjurholm	2,351	1,307	1.80	0	0
Nordmaling	7,050	1,231	5.73	1	1
Norsjö	3,917	1,739	2.25	1	1
Robertsfors	6,746	1292	5.22	1	1
Vindeln	5,480	2,630	2.08	1	1
Vännäs	9,041	530	17.06	1	1
Lycksele	12,204	5,518	2.21	1	3
Skellefteå	76,219	6,802	11.20	1	3
Umeå	133,112	2,317	56.47	1	5
West area	22,398	31,299	0.72	7	7
Dorotea	2,349	2,764	0.85	1	1
Malå	3,003	1,598	1.88	1	1
Sorsele	2,397	7,367	0.33	1	1
Storuman	5,634	7,299	0.77	2	2
Vilhelmina	6,281	8,047	0.78	1	1
Åsele	2,734	4,223	0.65	1	1
All municipalities	278,518	54,664	5.10	15	23

Supplementary Table 2: Categorical variables used in linear modelling

**Variable name**	**Description**	**Categories**
Age	Age of the patient	60+ = 1<br>0-59 = 0
Gender	Gender of the patient	Women = 1<br>Men = 0
Alarm location	Type of district where the alarm occurred	Urban districts = 1<br>Suburban or Rural districts = 0
Season	Season when the alarm call is made	November to April = 1<br>May to October = 0
Day of the week	Day of the week when the alarm call is made	Saturday or Sunday = 1<br>Other = 0
Time of the day	Time of the day when the alarm call is made	8:00 - 21:00 = 1<br>21:00 - 8:00 = 0

Supplementary Table 3: Proportions of priority-1 alarms in different DeSO district types together with total number of alarms for different demographic groups, based on dataset 1. Age is split into two categories: '0-59' and '60+'.

District	All			0-59			60+		
	All	Women	Men	All	Women	Men	All	Women	Men
Rural	0.23	0.20	0.26	0.23	0.22	0.25	0.23	0.20	0.26
Suburban	0.10	0.10	0.11	0.08	0.08	0.08	0.11	0.10	0.12
Urban	0.67	0.70	0.63	0.69	0.70	0.67	0.66	0.70	0.62
All	11,764	5,784	5,980	4,693	2,339	2,354	7,071	3,445	3,626

Supplementary Table 4: Alarm incidences for different areas and age groups. Succeeding age groups with a significant increase (decrease) in alarm incidence are denoted by '*', where the number of stars corresponds to different p-values: * ($p < 0.05$), ** ($p < 0.01$), and *** ($p < 0.001$). For example, the alarm incidence in the East area for people of age 0-59 was 0.035*, here the * indicates that the observed alarm incidence was significantly higher than for the age group 40-49.

Area	Age group									
	0-9	10-19	20-29	30-39	40-49	50-59	60-69	70-79	80-89	90+
East area	0.025	0.020	0.033***	0.029	0.026	0.035*	0.052***	0.092***	0.165***	0.308***
Lycksele	0.010	0.022**	0.021	0.028	0.020	0.034*	0.038	0.103***	0.203***	0.222
Skellefteå	0.017	0.021*	0.028**	0.022*	0.024	0.031**	0.042***	0.083***	0.177***	0.279***
Umeå	0.017	0.017	0.022***	0.020	0.018	0.027***	0.043***	0.088***	0.180***	0.280***
West area	0.025	0.024	0.038**	0.030	0.032	0.042	0.041	0.095***	0.144***	0.223***
All areas	0.018	0.020	0.025***	0.023*	0.022	0.031***	0.043***	0.089***	0.174***	0.274***

Supplementary Table 5: Differences in alarm incidence between men and women (men's incidence - women's incidence) for different areas and age groups. The number of stars corresponds to different p-values: * ($p < 0.05$), ** ($p < 0.01$), and *** ($p < 0.001$).

Area	Age group									
	0-9	10-19	20-29	30-39	40-49	50-59	60-69	70-79	80-89	90+
East area	0.006	-0.006	-0.009	-0.020***	-0.011*	0.008	0.008	0.013	0.061***	0.076
Lycksele	-0.006	-0.005	-0.009	0.007	0.007	0.009	0.010	0.055***	0.096**	0.114
Skellefteå	0.003	-0.010**	-0.007*	-0.003	-0.002	0.007*	0.016***	0.029***	0.026*	0.016
Umeå	0.006**	-0.009***	-0.003	-0.007***	-0.005*	0.011***	-0.004	0.017**	0.021*	0.038
West area	-0.002	-0.004	-0.018*	0.010	0.013	0.011	0.009	0.028**	0.063***	0.186***
All areas	0.004**	-0.008***	-0.005**	-0.006***	-0.003	0.010***	0.006**	0.023***	0.037***	0.060***

Supplementary Table 6: Median of the process times (minute) for priority-1 alarms in different areas and DeSO districts. Some row values are missing due to the fact that there are no suburban (type B) DeSO districts in Lycksele and West area.

Area	District	Response time	Dispatch time	Preparation time	Travel time
East Area	Rural	27.51	3.88	1.23	21,22
	Suburban	24.48	3.40	0.22	19.63
	Urban	13.97	3.63	1.27	6.32
	All	22.42	3.73	1.25	16.43
Lycksele	Rural	30.30	3.08	1.66	22.93
	Suburban	-	-	-	-
	Urban	9.87	3.38	1.58	4.38
	All	11.51	3.31	1.61	5.13
Skellefteå	Rural	29.23	3.93	1.07	22.95
	Suburban	23.57	3.75	0.97	18.28
	Urban	10.73	3.58	0.97	5.42
	All	14.62	3.68	0.98	8.30
Umeå	Rural	22.82	3.60	0.92	17.58
	Suburban	19.28	3.58	1.05	13.90
	Urban	12.13	3.48	0.93	7.14
	All	13.57	3.50	0.95	8.20
West Area	Rural	30.55	3.87	2.68	22.22
	Suburban	-	-	-	-
	Urban	12.50	3.55	2.63	4.83
	All	18.71	3.75	2.68	11.55

Supplementary Table 7: Median response times, dispatch times, preparation times, travel times and estimated travel times for demographic groups defined by age, gender, and DeSO districts: rural districts (A), suburban districts (B), urban districts (C) and all districts (All). Here, δ denotes difference in the median process times between different age groups, where the number of stars corresponds to different p-values of Mann-Whitney U test: * ($p < 0.05$), ** ($p < 0.01$), and *** ($p < 0.001$).

Process time	District	All				Men			Women		
		All	0-59	60+	δ	0-59	60+	δ	0-59	60+	δ
Response time	Rural	27.47	25.54	28.22	-2.68***	26.03	28.90	-2.87***	24.93	27.47	-2.53*
	Suburban	21.16	20.23	21.43	-1.19**	19.83	21.40	-1.57*	20.98	21.44	-0.47*
	Urban	11.67	11.17	12.02	-0.85***	10.83	11.98	-1.15***	11.48	12.03	-0.55***
	All	14.62	13.85	15.23	-1.38***	13.83	15.99	-2.16***	13.87	14.60	-0.73***
Dispatch time	Rural	3.80	3.23	4.10	-0.88***	3.12	4.11	-0.99***	3.30	4.10	-0.80***
	Suburban	3.63	3.10	3.83	-0.73***	2.83	3.82	-0.99***	3.40	3.83	-0.43*
	Urban	3.53	3.00	3.80	-0.80***	2.78	3.63	-0.85***	3.21	3.93	-0.73***
	All	3.60	3.07	3.87	-0.80***	2.88	3.78	-0.90***	3.23	3.97	-0.73***
Preparation time	Rural	1.27	1.27	1.27	0.00	1.37	1.31	0.06	1.13	1.20	-0.07
	Suburban	0.98	0.98	0.98	0.00	0.98	0.98	0.00	1.00	0.98	0.02
	Urban	1.03	1.02	1.03	-0.01	1.03	1.06	-0.03	1.01	1.03	-0.02
	All	1.07	1.05	1.07	-0.02	1.07	1.10	-0.03	1.03	1.05	-0.02
Travel time	Rural	21.00	19.88	21.57	-1.68**	20.35	22.15	-1.80**	19.67	20.58	-0.91
	Suburban	15.40	15.28	15.53	-0.25	14.76	16.08	-1.32	15.43	15.08	0.35
	Urban	6.17	6.15	6.20	-0.05	6.15	6.30	-0.15	6.12	6.12	0.00
	All	8.58	8.03	8.92	-0.88***	8.16	9.83	-1.67***	7.95	8.20	-0.25
Estimated travel time	Rural	19.41	18.20	20.43	-2.23***	18.16	21.47	-3.31***	18.27	18.89	-0.62
	Suburban	13.96	13.58	14.11	-0.53	13.51	14.49	-0.98	13.62	13.74	-0.12
	Urban	3.73	3.73	3.72	0.01	3.75	3.92	-0.17	3.72	3.55	0.17
	All	5.95	5.40	6.25	-0.85***	5.52	7.40	-1.88***	5.27	5.54	-0.27

Supplementary Table 8: Median response times, dispatch times, preparation times, travel times and estimated travel times for demographic groups defined by age, gender, and DeSO districts: rural districts (A), suburban districts (B), urban districts (C) and all districts (All). Here, δ denotes difference in the median process times between different gender groups, where the number of stars corresponds to different p-values of Mann-Whitney U test: * ($p < 0.05$), ** ($p < 0.01$), and *** ($p < 0.001$).

Process time	District	All				0-59			60+		
		All	Men	Women	δ	Men	Women	δ	Men	Women	δ
Response time	Rural	27.47	27.92	26.70	1.22**	26.03	24.93	1.10	28.90	27.47	1.43**
	Suburban	21.16	20.87	21.28	-0.42	19.83	20.98	-1.14	21.40	21.44	-0.04
	Urban	11.67	11.53	11.82	-0.28	10.83	11.48	-0.65*	11.98	12.03	-0.05
	All	14.62	15.02	14.28	0.73***	13.83	13.87	-0.03	15.99	14.60	1.39***
Dispatch time	Rural	3.80	3.77	3.84	-0.08	3.12	3.20	-0.18	4.11	4.10	0.01
	Suburban	3.63	3.53	3.73	-0.21	2.83	3.40	-0.58	3.82	3.83	-0.01
	Urban	3.53	3.35	3.72	-0.37***	2.78	3.21	-0.43***	3.63	3.93	-0.30***
	All	3.60	3.47	3.75	-0.28***	2.88	3.23	-0.35***	3.78	3.97	-0.19**
Preparation time	Rural	1.27	1.33	1.18	0.15**	1.37	1.13	0.24*	1.31	1.20	0.11*
	Suburban	0.98	0.98	0.98	0.00	0.98	1.00	-0.02	0.98	0.98	0.00
	Urban	1.03	1.05	1.02	0.03*	1.03	1.01	0.02	1.06	1.03	0.03
	All	1.07	1.08	1.05	0.03***	1.07	1.03	0.04*	1.10	1.05	0.05**
Travel time	Rural	21.00	21.53	20.22	1.31**	20.35	19.67	0.68	22.15	20.58	1.57**
	Suburban	15.40	15.88	15.18	0.70	14.76	15.43	-0.67	16.08	15.08	1.00
	Urban	6.17	6.25	6.12	0.13	6.15	6.12	0.03	6.30	6.12	0.18
	All	8.58	9.13	8.10	1.03***	8.16	7.95	0.21	9.83	8.20	1.63***
Estimated travel time	Rural	19.41	20.10	18.53	1.57**	18.16	18.27	-0.11	21.47	18.89	2.58**
	Suburban	13.96	14.29	13.65	0.64	13.51	13.62	-0.11	14.49	13.74	0.75
	Urban	3.73	3.85	3.62	0.23	3.75	3.72	0.03	3.92	3.55	0.37
	All	5.95	6.43	5.41	1.02***	5.52	5.27	0.25	7.40	5.54	1.86***

Supplementary Table 9: Mean preparation times for demographic groups defined by age, municipality groups: large municipalities (Lycksele, Nordmaling, Robertsfors, Skellefteå, Umeå, Vännäs), small municipalities (Bjurholm, Dorotea, Malå, Norsjö, Storuman, Sorsele, Vilhelmina, Vindeln, Åsele), and DeSO districts: Non-urban districts (A and B), urban districts (C) and all districts. Here, δ denotes difference in the mean preparation times between different age groups, where the number of stars corresponds to different p-values of Welch's t-test: * ($p < 0.05$), ** ($p < 0.01$), and *** ($p < 0.001$).

Process time	District	All				Large municipalities			Small municipalities		
		All	0-59	60+	δ	0-59	60+	δ	0-59	60+	δ
Preparation time	Non-urban	1.43	1.44	1.43	0.01	1.17	1.15	0.02	2.50	2.47	0.03
	Urban	1.20	1.17	1.22	-0.05	1.05	1.06	-0.01	2.48	2.39	0.09
	All	1.28	1.26	1.29	-0.03	1.08	1.09	-0.01	2.49	2.43	0.06

Supplementary Table 10: Mean preparation times for demographic groups defined by gender, municipality groups: large municipalities (Lycksele, Nordmaling, Robertsfors, Skellefteå, Umeå, Vännäs), small municipalities (Bjurholm, Dorotea, Malå, Norsjö, Storuman, Sorsele, Vilhelmina, Vindeln, Åsele), and DeSO districts: Non-urban districts (A and B), urban districts (C) and all districts. Here, δ denotes difference in the mean preparation times between different gender groups, where the number of stars corresponds to different p-values of Welch's t-test: * ($p < 0.05$), ** ($p < 0.01$), and *** ($p < 0.001$).

Process time	District	All				Large municipalities			Small municipalities		
		All	Men	Women	δ	Men	Women	δ	Men	Women	δ
Preparation time	Non-urban	1.43	1.49	1.36	0.13**	1.19	1.12	0.07	2.48	2.48	0.00
	Urban	1.20	1.23	1.18	0.05	1.06	1.05	0.01	2.51	2.33	0.18
	All	1.28	1.32	1.24	0.08***	1.11	1.07	0.04	2.49	2.39	0.10

Supplementary Table 11: Predicted coefficients together with corresponding p-value and 95%-confidence interval for parametric multivariate regression with the response variables dispatch time, preparation time, and travel time and the six binary variables age, gender, alarm location, season, day of the week, and time of the day. The number of stars corresponds to different p-values: * ($p < 0.05$), ** ($p < 0.01$), and *** ($p < 0.001$).

Factor	Response	Estimated coefficient	95%-confidence interval	
			Lower bound	Upper bound
Intercept	Dispatch time	4.09***	3.88	4.31
	Preparation time	1.85***	1.79	1.92
	Travel time	21.11***	20.61	21.62
Age	Dispatch time	0.61***	0.46	0.76
	Preparation time	0.09***	0.05	0.13
	Travel time	0.62***	0.28	0.96
Gender	Dispatch time	0.28***	0.13	0.42
	Preparation time	-0.06**	-0.10	0.01
	Travel time	-0.76***	-1.09	0.42
Alarm location	Dispatch time	-0.32***	-0.48	-0.17
	Preparation time	-0.23***	-0.27	-0.18
	Travel time	-13.28***	-13.64	-12.92
Season	Dispatch time	0.22**	0.07	0.36
	Preparation time	0.11***	0.06	0.15
	Travel time	0.70***	0.36	1.03
Day of the week	Dispatch time	-0.25**	-0.41	-0.10
	Preparation time	0.08***	0.03	0.13
	Travel time	-0.06	-0.42	0.31
Time of the day	Dispatch time	0.07	-0.09	0.22
	Preparation time	-0.77***	-0.81	0.72
	Travel time	0.04	-0.33	0.40

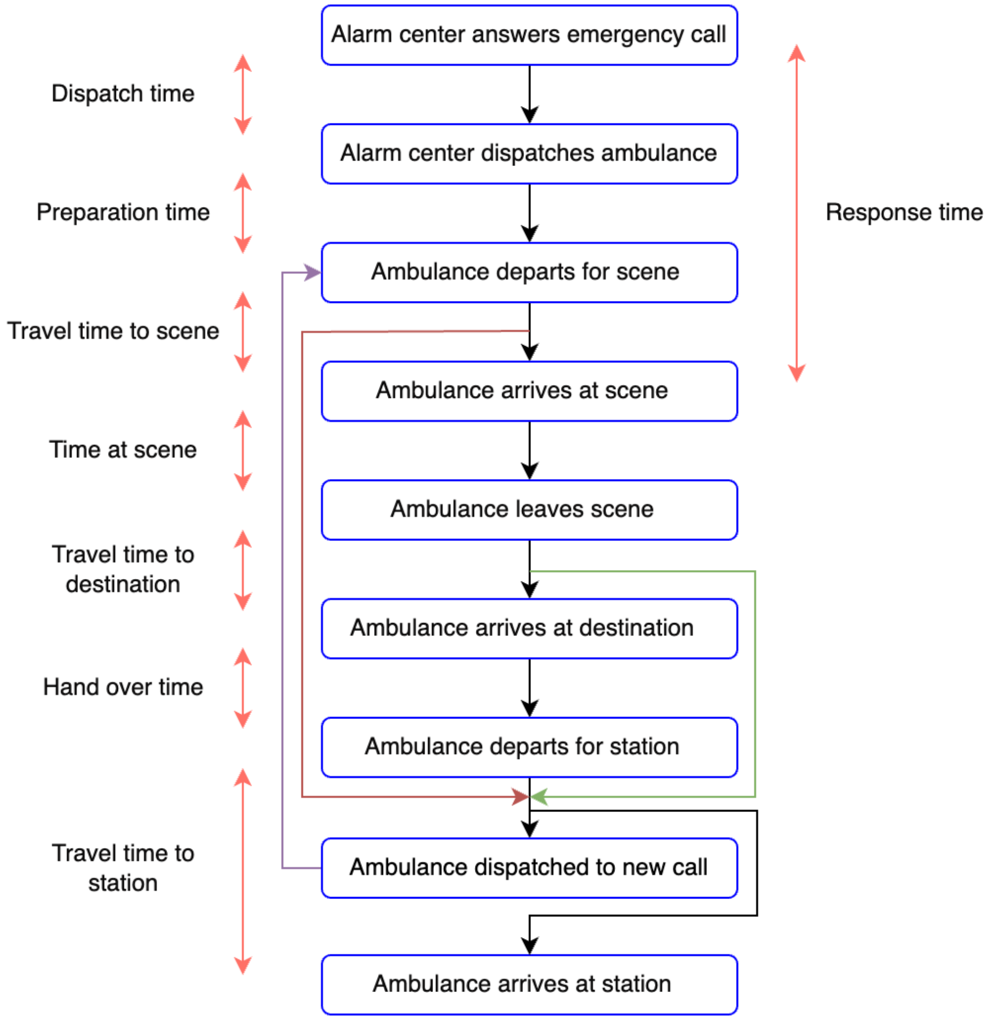

Supplementary Fig. 1: Flowchart of the ambulance dispatch and service process

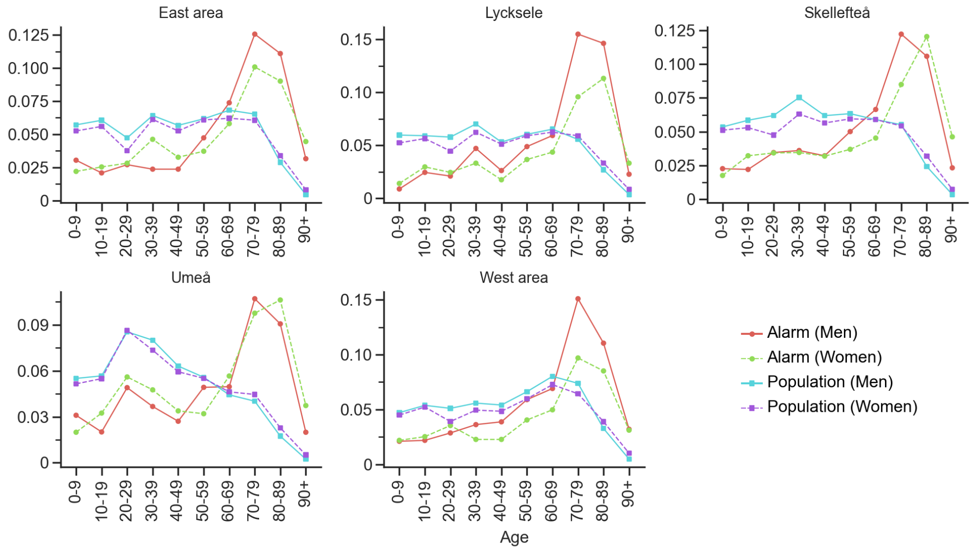

Supplementary Fig. 2: Fraction of priority-1 alarms by gender across age groups in five areas of Västerbotten County (men: green solid line; women: red dashed line). The corresponding population distribution is shown by age group (men: blue solid line; women: purple dashed line). In both cases, the male and female proportions sum to 1.

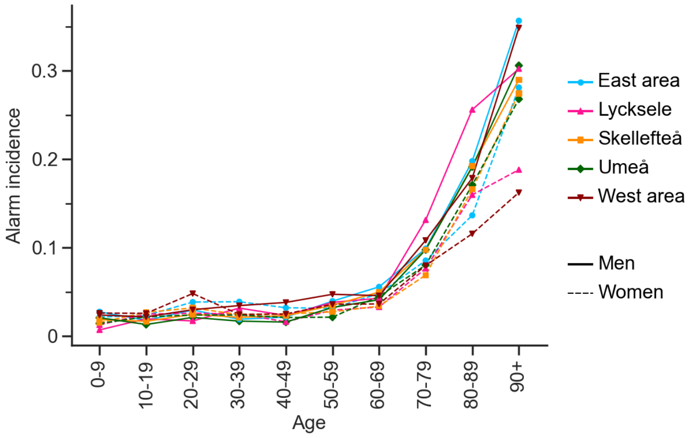

Supplementary Fig. 3: The alarm incidence for men (solid) and women (dashed) for different age groups and areas: East area (blue), Lycksele (pink), Skellefteå (yellow), Umeå (green), and the West area (brown).

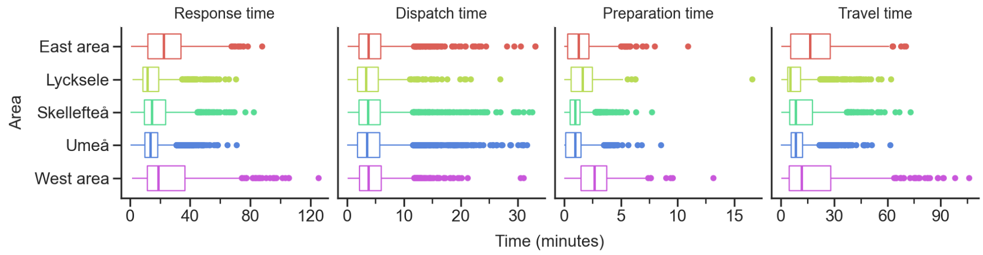

Supplementary Fig. 4: The box plot of time components of response time for priority-1 alarms in different areas. The box plot shows the first quartile, median, and third quartile of the process times. The whiskers extend to the most extreme data points within 1.5 times the interquartile range from the box. The points outside the whiskers are considered as outliers.

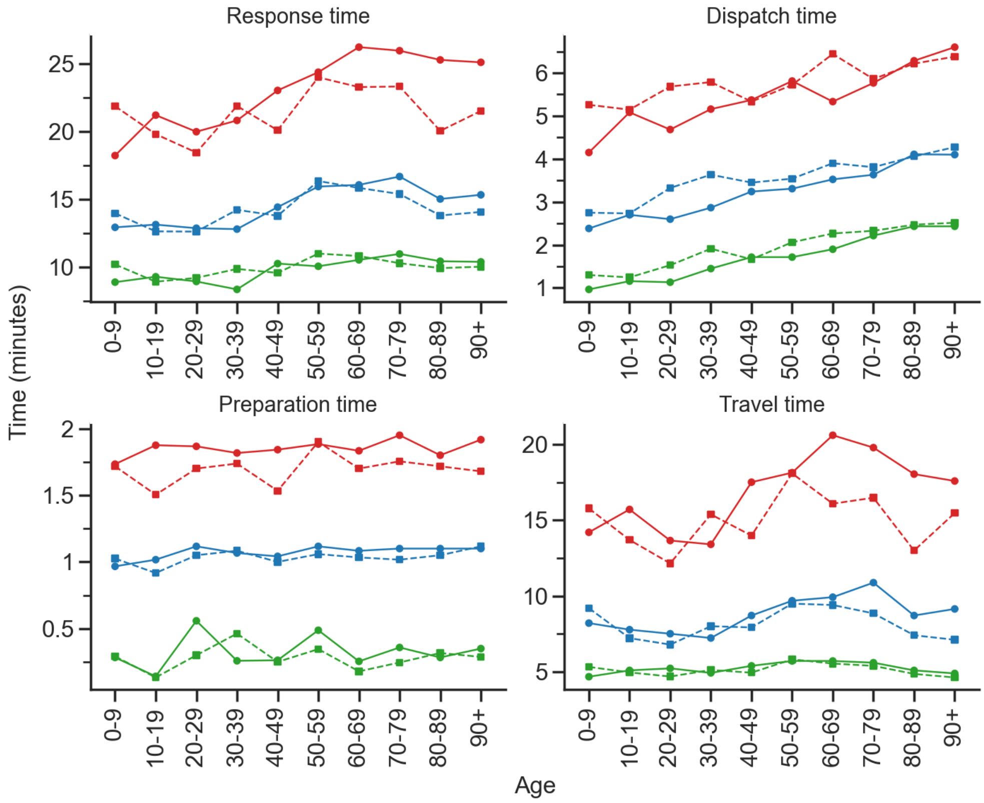

Supplementary Fig. 5: The first quartile (red), median (blue), and third quartile (green) of process times for priority-1 alarms in different demographic groups (men: solid, women: dashed).
